# Systematic evaluation of written health information on PSA based screening in Germany

**DOI:** 10.1371/journal.pone.0220745

**Published:** 2019-08-08

**Authors:** Simone Beck, Birgit Borutta, Ulla Walter, Maren Dreier

**Affiliations:** Institute of Epidemiology, Social Medicine and Health Systems Research, Hannover Medical School, Hannover, Germany; University of Toronto, CANADA

## Abstract

**Background:**

Prostate-specific antigen (PSA) based screening for early detection of prostate cancer is common although it is associated with both benefits and potential harms (e.g., the risk of overdiagnosis). Evidence-based health information could help individuals make informed decisions about whether to undergo PSA testing or not. This evaluation aimed to determine whether the written health information materials available in Germany provide appropriate information for informed decision-making on PSA based screening.

**Methods:**

A list of criteria was developed and used to systematically assess the quality of information on the benefits and harms of prostate cancer screening included in written health information materials. Fourteen information materials identified by information requests and online searches were evaluated independently by two of three reviewers. Consensus was achieved with a third reviewer.

**Results:**

Of the 14 information materials evaluated, 10 (71%) list the ability to reduce the absolute risk of death from prostate cancer as a benefit of PSA testing, 9 (64%) point out the risks of follow-up diagnostics, 13 (93%) describe the risks of the available prostate cancer treatments, and all 14 specify the risk of overdiagnosis. The minority provide numerical data on benefits and risks. Partially mismatched framing was identified in four cases: two information materials report only the relative frequencies of benefits, and two report only the absolute frequencies of harms. Half of the materials encouraged participation using downplaying or frightening language.

**Conclusions:**

The majority of health information materials in Germany describe the benefits and harms of PSA based screening, including overdiagnosis, but often lack adequate balance, neutrality and numbers.

## Introduction

Cancer screening is associated with a great potential for ethical conflicts because it exposes many individuals to risks and stresses in order to provide a few individuals the benefits of early detection [1, 2]. Prostate cancer (PCa) is the most common cancer and the second leading cause of death from cancer in the German male population [3]. For early detection of PCa, German statutory health insurances (compulsory; 85% of the German population are members) offer biennially from the age of 45 years an inspection and examination of the external genitalia, a digital rectal examination, and counselling. Prostate-specific antigen (PSA) based screening is not covered by German statutory health insurances, but must be paid out-of-pocket. The German S3-Guideline [4] recommends to inform individuals of 45 years or older with an expected life-expectancy of 10 years or more about the benefits and harms of PSA-testing only if they explicitly ask to be informed about PCa based screening.

The benefit of the PSA test in terms of reducing mortality is currently a matter of controversial debate. Two important randomized controlled trials (RCT) reported different effects of PSA based screening on PCa mortality [5, 6]. Meta-analyses of all RCTs including the recently published British cluster RCT showed that it produced no reduction in PCa mortality or overall mortality [7–9]. Besides having limited benefit, PCa screening is accompanied by substantial risks, including overdiagnosis (prevalence: 30–50%) and false-positive results, which can lead to unnecessary biopsies and prostate surgery [6, 8, 10–15].

These potential risks underline the importance of adequately informing individuals of the benefits and harms of PSA testing to enhance their ability to make an informed decision for or against prostate cancer screening [16, 17]. Written health information that meets the criteria for evidence-based health information can support informed decision-making. There is evidence that evidence-based decision aids for prostate cancer screening can increase one’s knowledge about PSA testing [17–21]. The definitions of evidence-based health information and DA are overlapping, the latter aiming implicitly or explicitly (with a special tool) to clarify one’s preferences and values [17]; in this article the terms are used interchangeably. Evidence-based health information should provide information that corresponds to the current state of scientific knowledge, is comprehensible and balanced, states both the potential benefits and risks, and explains the reliability of scientific evidence concerning a test or procedure [22, 23]. Moreover, it must depict the risks and benefits of a test or procedure in a balanced manner, and must not be worded so as to sway the reader in a certain direction [24]. Evidence-based health information must explicitly mention that the decision to not take a test is as appropriate as the decision to take the test [25], and it should not attempt to motivate individuals to take the test by downplaying harms or by generating fear [26]. Of great importance is a quantitative presentation of information about the frequencies of benefits and risks, preferably using absolute numbers [23, 27]. It is also crucial to avoid mismatched framing [28]. For example, the authors of some information materials report benefits in terms of percentages of relative risk reduction, which are generally high, while at the same time reporting harms in terms of absolute risk numbers, which are usually low, resulting in a distorted/biased picture in favor of taking the test.

Previous studies found deficiencies in written health information materials on PSA based screening in terms of incomplete information on the harms, or an unbalanced presentation of the benefits and harms [29–31]. The aim of this study was to evaluate whether German written health information materials provide balanced, unbiased and comprehensible information on the harms and benefits of PSA based screening.

## Materials and methods

### Study design

To evaluate the appropriateness of information on PSA based screening in Germany, available information leaflets and booklets on PSA testing were identified and assessed using an adapted, comprehensive list of criteria. The study protocol was approved by the ethics committee of the Hannover Medical School (Application No. 3573–2017).

### Development of the list of criteria and manual

Selection and phrasing of the criteria were conducted analogously to a comprehensive list of criteria for information material on colorectal cancer screening, which was developed by a systematic literature search involving an expert panel [32]. The original list was successfully used to evaluate written and online-based information materials on colorectal cancer screening in 2014 [33]. For the current review of information on PSA based screening, we added modified criteria for benefits and risks as well as criteria for neutrality and balance of content and new criteria for the risk of overdiagnosis. These criteria were used for multidimensional assessments encompassing questions on whether certain content was included in the material (yes/no), whether the content was correct (yes/no/unclear), and whether information on the strength of evidence was provided. In the case of content with potentially numerical information (e.g., on risks, benefits, specificity and sensitivity), the reviewer indicated whether the information was presented in a textual, numerical, graphical or tabular format, and specified how the numerical data were presented (e.g., as percentages, absolute frequencies or natural frequencies with or without the same denominator).

The list of 47 criteria that we developed to assess written health information materials on PSA based screening is divided into five main categories and eleven subcategories (Table 1). The categories mainly encompass patient-oriented outcomes like the individual risk of getting or dying from prostate cancer, the effect of screening on life expectancy, and the possible side-effects of screening. The complete list is provided in the additional file S2 Appendix.

**Table 1:** List of 47 assessment criteria by categories (n = 5) and subcategories (n = 11).

Test characteristics (12)	Risks of disease and risk of death (2)	Benefits of the test (7)	Risks of the test (23)	Neutrality and balance (3)
Test quality (8)		PCa-specific mortality (4)	Overdiagnosis (3)	
Aim of the test (2)		Overall mortality (3)	Risks of follow-up diagnostics (biopsy) (8)	
Procedure depending on test result (2)			Risks of PCa treatment (4)	
			Psychological distress caused by false positive results (4)	
			Psychological distress caused by PCa diagnosis (2)	
			Other (2)	

The number of criteria is shown in parentheses; PCa, prostate cancer.

An answer manual was developed to help the reviewers make correct assessments and to minimize the subjectivity of ratings. The manual was based on the current evidence on PCa screening extracted from documents found in a systematic literature search of nine relevant electronic databases (including the PubMed, Embase, and Cochrane databases), conducted in April 2013 using the search interface of the German Institute of Medical Documentation and Information (Additional file S1 Appendix). The search was restricted to articles in English or German published from 1/2003 to 4/2013, a period that covers relevant evidence on PCa screening. The identified documents were selected by two independent researchers based on the relevance of content in stepwise fashion, proceeding from title to abstract to full text. Any discrepancies were decided by consensus with a third reviewer. The full texts of the included documents were analyzed and all relevant information was extracted and transferred to the manual. Evidence levels were assigned according to the recommendations of the Oxford Centre for Evidence-Based Medicine Levels of Evidence Working Group 2011 [34].

### Identification of information materials

In March 2014, we received relevant written information materials (booklets and leaflets) by directly corresponding via email with the most important stakeholders in early detection of (prostate) cancer in Germany, including the ten largest statutory health insurance companies, the National Association of Statutory Health Insurance Funds, other associations like the Federal Joint Committee (G-BA), scientific research institutes, expert associations of the Association of the Scientific Medical Societies (AWMF), German institution of PCa support groups (Bundesverband Prostatakrebs Selbsthilfe e.V.), and healthcare providers. Previously, an online search with the search engine Google using relevant terms (PSA test, PCa screening, PSA screening, PSA, preventive examination prostate cancer, health information, booklet, leaflet) had been conducted in June 2013. The first 200 results were reviewed. The identified websites and their sub-websites were searched for relevant information materials. Only those booklets and leaflets addressing individuals with an average risk of PCa were included in the study. Information materials for individuals diagnosed with prostate cancer, regional print media, or media published by pharmaceutical companies were excluded.

### Assessment

The 14 information materials included in the study were assessed independently by two out of three reviewers (SB, MD, BB). Any discrepancies were discussed and resolved by consensus between the three reviewers. Analyses were restricted to qualitative comparisons because the frequencies were too small for sound statistical analysis. An interrater reliability was not analyzed.

## Results

Twenty-one (67.7%) of the 31 healthcare stakeholders contacted responded to our email requests and provided a total of nine information materials, consisting of leaflets and booklets. Our online search yielded 11 potential information materials, 7 of which met the inclusion criteria (2 were identical to materials acquired by email request). A total of 6 leaflets and 8 booklets were included in the analysis. The identified leaflets and booklets spanned 2 to 87 pages and were published from 2001 to 2014. In some of cases, the publishing date was unclear. Most of the included health information materials were published by health insurance companies, foundations and scientific societies.

### Benefits

**Fig 1:**
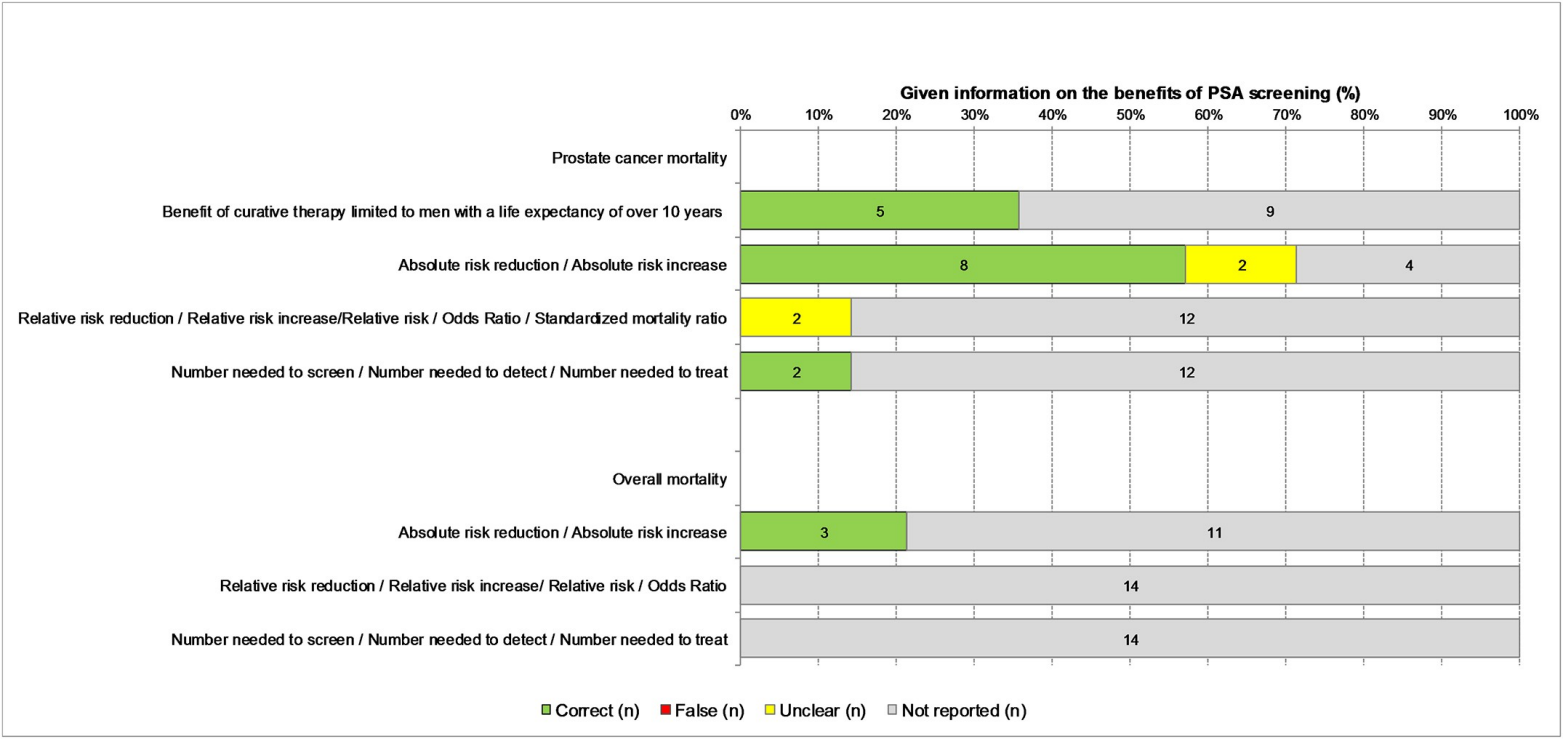
Information on the benefits of PSA based screening (n = 14 information materials).

The results of our assessment of the quality of information on the benefits of PSA based screening are shown in Fig 1. Approximately 71% (n = 10) of the 14 information materials contain statements reporting that PCa-specific mortality can be reduced through regular PSA testing (example: “PSA-based early detection and treatment of prostate cancer can theoretically reduce the mortality of prostate cancer”). Most of the information materials reflect the results of the Prostate, Lung, Colorectal, and Ovarian (PLCO) Cancer Screening Trial and the European Randomized Study of Screening for Prostate Cancer (ERSPC), which are presented as short texts and/or numbers [5, 9, 10, 35]. Moreover, 21% (n = 3) describe the impact of PSA testing on overall mortality (sample statement: “However, none of the studies showed that individuals in the PSA test group actually lived longer”).

### Risks

**Fig 2:**
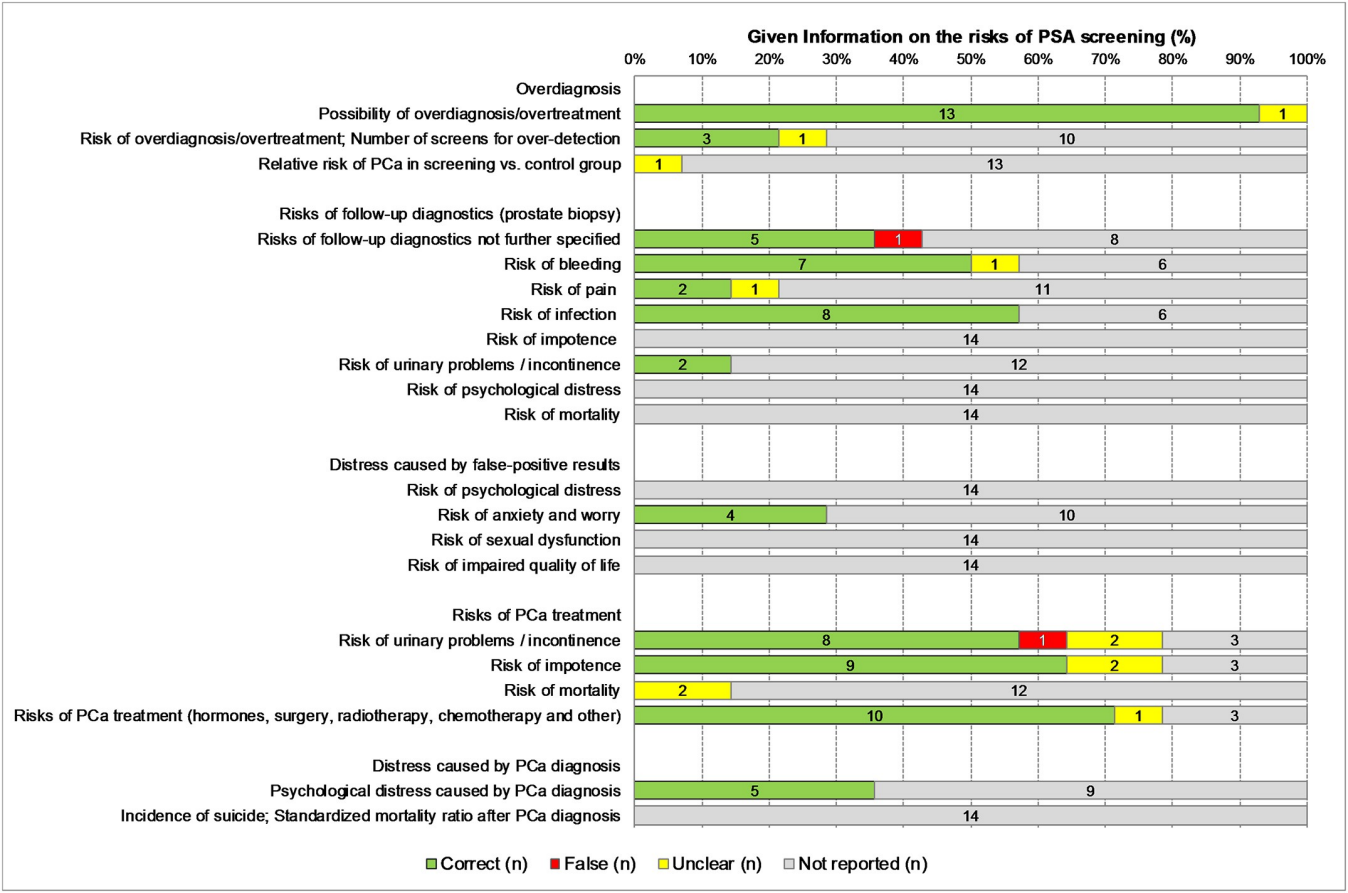
Information on the risks of PSA based screening (n = 14 information materials).

All 14 leaflets and booklets studied inform of the risk of overdiagnosis (Fig 2). They contain statements such as “At the same time, there is a much higher risk of overtreatment; tumors may be detected and possibly treated which would have never caused you any harm. Possible consequences of this overtreatment include impotence and incontinence.” Hence, they provide evidence that overdiagnosis and overtreatment are important risks of PSA testing. About 64% (n = 9) of the 14 information materials outline the risks of biopsy following a positive PSA test result, and 57% provide detailed information on the risk of bleeding (n = 8) and infection (n = 8), but less than one-third (n = 4) describe the risk of psychological distress caused by false-positive results. Approximately one-third (n = 5) do not mention follow-up diagnostic tests and the risks associated with them, while almost all of the materials (n = 13; 93%) elucidate the risks of treatment in case of prostate cancer diagnosis. The scope of clarification extends from short statements (example: “This form of treatment has side effects […]”) to detailed information on the available treatment options. More than one-third (n = 5) of the 14 information materials indicate the risk of psychological distress after PCa diagnosis (example: “The diagnosis causes anxiety and worry in the affected men and their families”).

### Numerical data

The minority of the 14 evaluated information materials (leaflets and booklets) provide numerical data on either the benefits (n = 6) or the risks (n = 8) of PSA based screening. Four (29%) describe the benefits of PSA testing in terms of absolute risk reduction (ARR), and two (14%) in terms of relative risk reduction (RRR) of PCa mortality. None of the information materials present both RRR and ARR statistics. Regarding the different potential risks, some report the absolute frequencies of overdiagnosis (n = 4), side-effects of prostate biopsy as a follow-up diagnostic test (n = 4), impotence and/or incontinence caused by PCa treatment (n = 3), and other report relative frequencies of overdiagnosis (n = 1) and incontinence after treatment (n = 1, resolved one year after surgery). None provide numerical data on the risk of pain and psychological distress. Some of the information materials exhibit partially mismatched framing: two report only the relative frequencies of benefits without numerical information on the harms, and two only the absolute frequencies of harms without numerical information on the benefits.

### Neutrality

**Fig 3:**
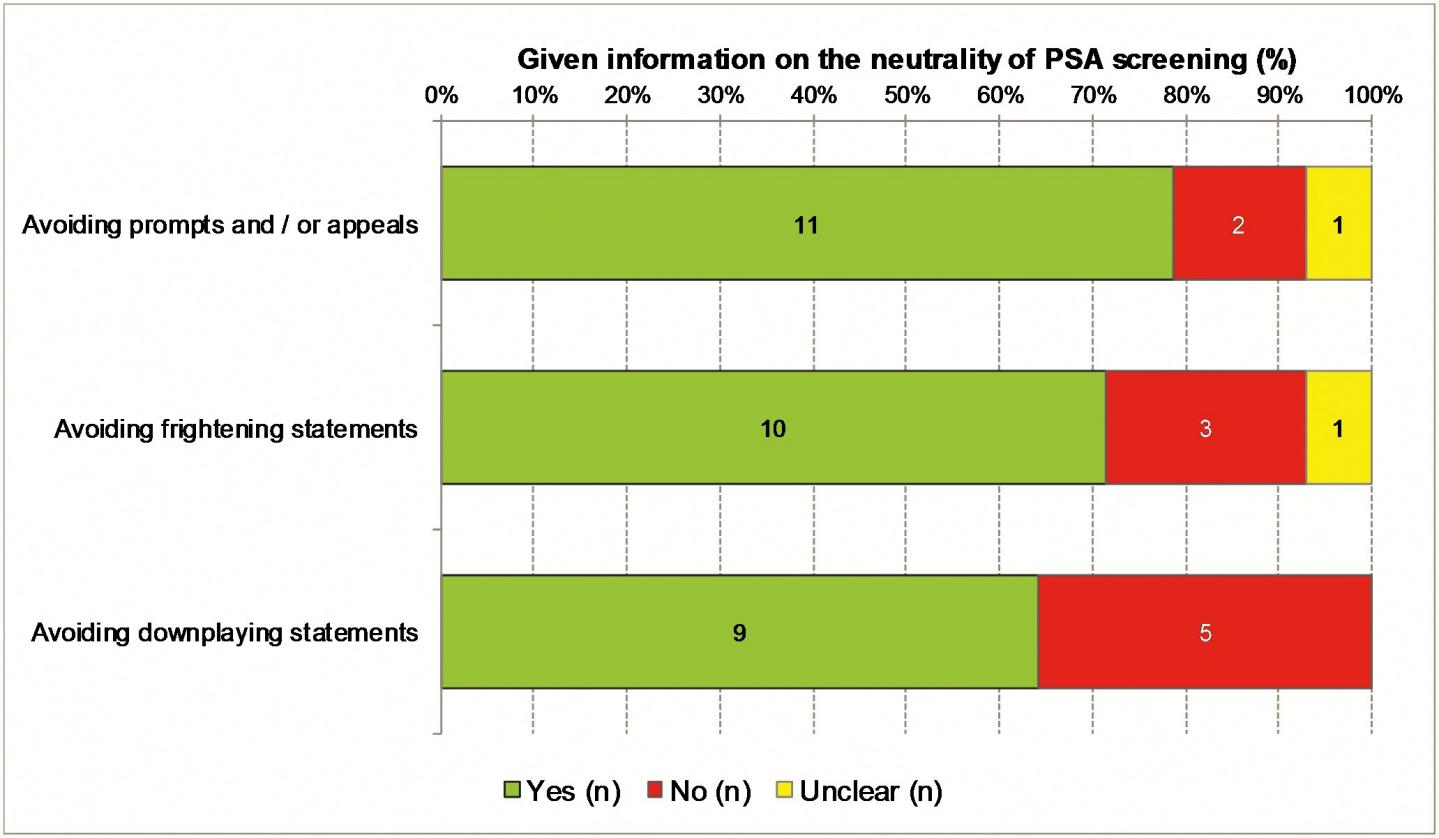
Information on the neutrality of PSA based screening (n = 14 information materials).

Approximately 79% percent (n = 11) of the 14 information materials did not use prompts and appeals, while 14% (n = 3) did not keep the neutrality principle. Of the three non-neutral information materials s, two promote screening with statements such as “…we encourage you to attend your prostate check early and regularly”, and one advises against taking the PSA test. Three (21%) of the information materials s contain frightening statements such as “Dying from prostate cancer is not the only thing that can happen. […] a long path of illness and suffering is not uncommon”. Downplaying statements such as “Given that a biopsy is a relatively harmless procedure, it appears reasonable to accept a slight risk of overdiagnosis” were found in five (36%). Seven (50%) met all the criteria for neutrality and balance of information (Fig 3).

## Discussion

Information materials that meet the criteria of evidence-based health information can empower individuals to make a properly informed decision about whether to undergo PSA based screening or not. The 14 information leaflets and booklets reviewed here using a comprehensive list of criteria, which was designed specifically for this purpose, exhibit deficits in the quality of information on the benefits of PSA testing, and most fail to mention its unknown effect on total mortality. In contrast, all of the information materials mention the risk of overdiagnosis, and most describe further risks based on the current scientific evidence. However, numerical data are generally lacking, and half of the information materialss fail to provide neutral and balanced information that neither encourages nor discourages PSA testing.

### Method used to evaluate the information

To our knowledge, there is no specific tool for the evaluation of information materials on PSA based screening that fully incorporates the current evidence-based health information recommendations. Existing generic tools such as DISCERN [36], Check-In-Instrument [37], International Patient Decision Aid Standards (IPDAS) Checklist [38], and IPDAS Instrument (IPDASi) [39, 40] focus on the type of information included, but not its correctness. Our modified tool considers specific patient-related outcomes when describing the potential benefits and harms of PSA based screening and verifies their validity [22, 24]. To support the reviewers, we created a manual providing correct sample answers to each criterion based on a systematic literature review. To ensure that the manual includes current evidence-based information, it must be continuously reviewed and regularly updated, e.g., biennially [24].

### Comparison with international studies

Three international studies had already evaluated information materials on the PSA test [29–31]. A study from Austria examined 17 information materials to determine whether the information contained in them is evidence-based and presented in a balanced manner based on defined evidence-based health information criteria from Steckelberg et al. [41]. The results revealed that the currently available information materials in Austria do not provide an adequate base for informed decision-making [31]. Another Austrian study evaluated the evidence-based nature, content and reporting of information on the benefits and risks of cancer screening (breast, colorectal, and cervical cancer and PCA screening) in 22 relevant information materials from Germany, Austria and Switzerland [29] using general criteria proposed by Bunge et al. [22]. Only five of the 22 information materials dealt with prostate cancer screening, and none satisfied all the quality criteria. Most did not meet the requirements for balanced reporting of the benefits and risks of screening. Direct comparison of these results with ours is not possible because of the use of different evaluation criteria, for example, specific screening-specific criteria were not used (e.g. the risk of overdiagnosis, risks of follow-up diagnostics (biopsy) or treatment), but only common criteria on the benefits and harms, and the correctness of information was not examined.

The third study from the Netherlands assessed 23 information materials on PSA testing from the USA, Canada, Australia and Europe [30]. Of the 23 leaflets and booklets studied, 17 (74%) outlined the risk of overdiagnosis, and 16 (70%) mentioned side effects of treatment in case of PCa diagnosis [30]. By comparison, all of our German materials assessed describe the risks of overdiagnosis, and 93% the risks of follow-up treatment. The better results for the German information materials can probably be attributed to the improved quality of health information over time: the Dutch study included materials printed from 2007 to 2009, whereas the German leaflets and booklets were published from 2009 to 2014. Moreover, the critical debate on the German National Cancer Plan included the concepts of informed choice and the provision of balanced and unbiased information on screening, which may have positively impacted the quality of health information materials in Germany [42].

### Challenge of informed decision making

Most (71%, n = 10) of the 14 leaflets and booklets assessed in this study provide information on the benefits and risks of PSA based screening, but only a few add the numerical data needed to give the reader a comprehensive picture of the dimensions of the benefits and risks [22]. Previous studies have shown that the benefits of PSA based screening are often overestimated [43]. None of the information materials evaluated here reported uncertainties of the results in terms of confidence intervals [22]. Some leaflets and booklets that actually report numerical data use absolute frequencies with unequal denominators, which might hamper comprehensibility [22]. After checking for mismatched framing as described previously [28], we found incomplete or partial mismatched framing in four information materials, which report only the relative frequencies of benefits or the absolute frequencies of harms, which might lead the reader to overestimate the benefits or underestimate the harms. We found that only half of the information materials maintain neutrality without downplaying or exaggerating benefits or risks. Nevertheless, this is more than the rate reported in a previous study of health information on colorectal cancer screening, in which merely 7% of the leaflets and one-third of the booklets and websites maintained neutrality [44]. We found two information materials that explicitly encourage the reader to undergo PSA testing and one that advises against the test. This clearly violates the modern principle of risk communication which requires health information drafters to inform readers in an open and unbiased manner [22, 24, 25, 41]. However, in comparison with previous studies [29–31, 33], it seems that the principles of informed decision-making are being increasingly implemented by more and more stakeholders in the German healthcare system.

While we could not determine whether the evaluated information materials do indeed support informed decision-making, several studies have found first evidence for the effectiveness of interventions to support informed decision-making. A computer-assisted telephone counseling decision aid enhanced PSA related knowledge and decision-making [45]. A study of Hispanic individuals showed that a community-based education program to promote informed decision-making for PSA testing increased knowledge about prostate cancer screening [46]. Decision aids for prostate cancer screening and evidence-based information can increase one’s knowledge about PSA testing [18–21, 47–50].

Information materials increasingly include explicit values clarification methods that support subjects to elicit their individual values and preferences. Our identified information materials did not include any explicit values clarification methods. Studies found that different values clarification methods like rating or ranking may produce different effects on the individual preferences and choices depending on its attributes and technique [51, 52]. Thus, simply adding a single item in our tool on the presence of such a method would neglect its quality. Instead, a specific tool to assess the values clarification method is needed, but to our knowledge, no one currently exists.

### Study limitations

This study is subject to certain limitations. Our list of assessment criteria, for example, is not a classical evaluation tool that allows for assessment and comparison of the quality of health information materials based on sum score calculations. We believe that weighting and prioritization of the criteria is needed for meaningful results. Simply adding up the number of criteria met implies that all of the criteria have equal weight and value. Thus, simple sum score calculation could lead to aggregation of the data and, possibly, to a loss of information because of single-criterion value losses [53]. In the future, researchers could explore evidence to weight the criteria in order to obtain a more valid overall evaluation. Furthermore, our list of criteria might not completely cover all possible outcomes for the benefits and risks of PSA based screening. Health information materials must not necessarily meet all of the criteria in order to provide individuals adequate information for informed decision-making. However, the scope of the evaluated information materials, ranging from short leaflets to more detailed booklets, seems to correlate with the detail of the content. Further research is needed to define the minimum information requirements for both short and comprehensive formats for health information materials. The latest materials we evaluated were from 2014, however, our findings of problems with neutrality, mismatched framing and lack of numerical data are relevant today and may help improve future information materials.

## Conclusions

There seems to be a national and international trend towards producing unbiased and balanced evidence-based health information to increase the implementation of informed decision-making on PSA based screening. The available health information materials identified and assessed in this study include less false, missing or misleading information than those in earlier studies. One notably positive finding is that all of the available information materials inform the reader of the important risk of overdiagnosis. However, some of the materials fail to maintain neutrality and instead recommend taking or not taking the PSA test; some provide outdated data, and the majority lack appropriate numerical data. There is still room to improve the neutrality of content by avoiding appeals as well as downplaying and frightening language. In the future, updated and new health information materials should include adequate and comprehensible numerical data according to the current recommendations. Moreover, researchers should examine whether health information materials on PSA based screening have the information quality needed to actually support informed decision-making by the target population and by individuals with different levels of education and health literacy.

## Supporting information

S1 Appendix Literature search strategy. (PDF)

S2 Appendix List of assessment criteria. (PDF)

## Abbreviations

ARR: Absolute risk reduction
ERSPC: European Randomized Study of Screening for Prostate Cancer
IPDAS: International Patient Decision Aid Standards
PCa: Prostate cancer
PLCO: Prostate, Lung, Colorectal and Ovarian cancer screening trial
PSA: Prostate-specific antigen
RCT: Randomized controlled trial
RRR: Relative risk reduction
