# Supplementary material for: Systematic evaluation of written health information on PSA based screening in Germany
Source: PLoS One. 2019 Aug 8;14(8):e0220745. doi: 10.1371/journal.pone.0220745 (PMC6687135; doi:10.1371/journal.pone.0220745)
Supplement: S1 Appendix — (PDF) [file pone.0220745.s001.pdf]

Literature search strategy

Table 1: Block building strategy

Block 1	R	Block 2	R	Block 3	R	Block 4	R
Prostat* (TITEL)	+++	PSA* (TITEL)	++	Früherkennung	+/-	Nutzen	+
		Prostate-specific antigen*(MeSH) (TITEL)	++	Screening (dt.)	++	Risik*	+++
				Screening (engl.)	+++	Risk*	+++
				Early diagnosis*	++	Benefit#	+
				Early detection*	++	Effect#	+
				Mass screening (MeSH)	+++	over-diagnosis	+/-
				Cancer screening (MeSH)	++	mortality	+++
				Screening program## (MeSH)	+++	Mortalität	+++
				Cancer screening test* (MeSH)	+	Überdiagnose/ Übertherapie	0
				Cancer early diagnosis (MeSH))	+	Adverse effect*	+++

R = relevance of the term based on the number of results (+++ >100.000 results, ++ 10.000-99.999 results, += 100-9.999 results, +/- 1-99 results, 0 no results), * = any number of letters, # = maximum of one letter, MeSH = Medical Subject Headings, TITEL = the term is searched in the title

The terms of each block were combined with OR.

The blocks were combined as follows: (1 OR 2) AND 3 AND 4“

Table 2: Included electronic data bases

Data base
MEDLINE
BIOSIS Previews
DAHTA-Datenbank
Deutsches Ärzteblatt
EMBASE
EMBASE Alert
gms
gms Meetings
SciSearch

Table 3: Inclusion and exclusion criteria

Inclusion criteria	Exclusion criteria
Benefits and/or harms/mortality of PSA screening	Asian or African populations
Studien mit nicht an Prostatakrebs erkrankten Männern	High-risk groups
European/American/Australian populations	Patients with PCa
Published 2003-2013	Editorials
Language German or English	Reply/letters/comments
Risks of biopsy	Active surveillance
	Only abstracts availble
