# Supplementary material for: Systematic evaluation of written health information on PSA based screening in Germany
Source: PLoS One. 2019 Aug 8;14(8):e0220745. doi: 10.1371/journal.pone.0220745 (PMC6687135; doi:10.1371/journal.pone.0220745)
Supplement: S2 Appendix — (PDF) [file pone.0220745.s002.pdf]

Title of Health information: ______________________________

Number of pages: _______

ID: ____________________

Name of reviewer: ____________________

Date: ____________________

	Criteria	Included?	Correct?	Kind of presentation?	Kind of numbers?	Strength of evidence indicated?	Quotes, Comments
**Test quality**							
**1**	General information of the test quality	❒ yes<br>❒ no	❒yes<br>❒ no<br>❒ unclear			❒ yes<br>❒ no<br>❒ Indication on missing evidence	
**2**	Sensitivity	❒ yes<br>❒ no	❒yes<br>❒ no<br>❒ unclear	❒ Text<br>❒ Number<br>❒ Diagram<br>❒Table	❒ Percentage<br>❒ Absolute frequency without reference value<br>❒ Natural frequency with equal reference value<br>❒ Natural frequency with different reference value	❒ yes<br>❒ no<br>❒ Indication on missing evidence	
**3**	Specificity	❒ yes<br>❒ no	❒yes<br>❒ no<br>❒ unclear	❒ Text<br>❒ Number<br>❒ Diagram<br>❒Table	❒ Percentage<br>❒ Absolute frequency without reference value<br>❒ Natural frequency with equal reference value<br>❒ Natural frequency with different reference value	❒ yes<br>❒ no<br>❒ Indication on missing evidence	
**4**	Frequency of a positive result	❒ yes<br>❒ no	❒yes<br>❒ no<br>❒ unclear	❒ Text<br>❒ Number<br>❒ Diagram<br>❒Table	❒ Percentage<br>❒ Absolute frequency without reference value<br>❒ Natural frequency with equal reference value<br>❒ Natural frequency with different reference value	❒ yes<br>❒ no<br>❒ Indication on missing evidence	
**5**	Positive predictive value	❒ yes<br>❒ no	❒yes<br>❒ no<br>❒ unclear	❒ Text<br>❒ Number<br>❒ Diagram<br>❒Table	❒ Percentage<br>❒ Absolute frequency without reference value<br>❒ Natural frequency with equal reference value<br>❒ Natural frequency with different reference value	❒ yes<br>❒ no<br>❒ Indication on missing evidence	
**6**	Frequency of a false-positive result	❒ yes<br>❒ no	❒yes<br>❒ no<br>❒ unclear	❒ Text<br>❒ Number<br>❒ Diagram<br>❒Table	❒ Percentage<br>❒ Absolute frequency without reference value<br>❒ Natural frequency with equal reference value<br>❒ Natural frequency with different reference value	❒ yes<br>❒ no<br>❒ Indication on missing evidence	
**7**	Negative predictive value	❒ yes<br>❒ no	❒yes<br>❒ no<br>❒ unclear	❒ Text<br>❒ Number<br>❒ Diagram<br>❒Table	❒ Percentage<br>❒ Absolute frequency without reference value<br>❒ Natural frequency with equal reference value<br>❒ Natural frequency with different reference value	❒ yes<br>❒ no<br>❒ Indication on missing evidence	
**8**	Frequency of a false-negative result	❒ yes<br>❒ no	❒yes<br>❒ no<br>❒ unclear	❒ Text<br>❒ Number<br>❒ Diagram<br>❒Table	❒ Percentage<br>❒ Absolute frequency without reference value<br>❒ Natural frequency with equal reference value<br>❒ Natural frequency with different reference value	❒ yes<br>❒ no<br>❒ Indication on missing evidence	
**Aim of the test**							
**9**	General information on the relevance of PSA screening	❒ yes<br>❒ no	❒yes<br>❒ no<br>❒ unclear			❒ yes<br>❒ no<br>❒ Indication on missing evidence	
**10**	Information on the significance of a single test (distortion due to infections, etc.)	❒ yes<br>❒ no	❒yes<br>❒ no<br>❒ unclear			❒ yes<br>❒ no<br>❒ Indication on missing evidence	
**Risk of disease and risk of death**							
**11**	Incidence of prostate cancer	❒ yes	❒yes	❒ Text	❒ Percentage	❒ yes	

	Criteria	Included?	Correct?	Kind of presentation?	Kind of numbers?	Strength of evidence indicated?	Quotes, Comments
		❒ no	❒ no<br>❒ unclear	❒ Number<br>❒ Diagram<br>❒Table	❒ Absolute frequency without reference value<br>❒ Natural frequency with equal reference value<br>❒ Natural frequency with different reference value	❒ no<br>❒ Indication on missing evidence	
**12**	Mortality of prostate cancer	❒ yes<br>❒ no	❒yes<br>❒ no<br>❒ unclear	❒ Text<br>❒ Number<br>❒ Diagram<br>❒Table	❒ Percentage<br>❒ Absolute frequency without reference value<br>❒ Natural frequency with equal reference value<br>❒ Natural frequency with different reference value	❒ yes<br>❒ no<br>❒ Indication on missing evidence	
**Benefits of PSA testing: mortality of PCa**							
**13**	Information that only men with a residual life expectancy of more than 10 years benefit from curative therapy	❒ yes<br>❒ no	❒yes<br>❒ no<br>❒ unclear	❒ Text<br>❒ Number<br>❒ Diagram<br>❒Table		❒ yes<br>❒ no<br>❒ Indication on missing evidence	
**14**	Absolute risk reduction/ absolute risk increase	❒ yes<br>❒ no	❒yes<br>❒ no<br>❒ unclear	❒ Text<br>❒ Number<br>❒ Diagram<br>❒Table	❒ Percentage<br>❒ Absolute frequency without reference value<br>❒ Natural frequency with equal reference value<br>❒ Natural frequency with different reference value	❒ yes<br>❒ no<br>❒ Indication on missing evidence	
**15**	Relative risk reduction/ relative risk excess / odds ratio / standardized mortality rate	❒ yes<br>❒ no	❒yes<br>❒ no<br>❒ unclear	❒ Text<br>❒ Number<br>❒ Diagram<br>❒Table	❒ Percentage<br>❒ Absolute frequency without reference value<br>❒ Natural frequency with equal reference value<br>❒ Natural frequency with different reference value	❒ yes<br>❒ no<br>❒ Indication on missing evidence	
**16**	Number needed to screen / number needed to detect / number needed to treat	❒ yes<br>❒ no	❒yes<br>❒ no<br>❒ unclear	❒ Text<br>❒ Number<br>❒ Diagram<br>❒Table	❒ Percentage<br>❒ Absolute frequency without reference value<br>❒ Natural frequency with equal reference value<br>❒ Natural frequency with different reference value	❒ yes<br>❒ no<br>❒ Indication on missing evidence	
**Benefits of PSA testing: overall mortality**							
**17**	Absolute risk reduction/ absolute risk increase	❒ yes<br>❒ no	❒yes<br>❒ no<br>❒ unclear	❒ Text<br>❒ Number<br>❒ Diagram<br>❒Table	❒ Percentage<br>❒ Absolute frequency without reference value<br>❒ Natural frequency with equal reference value<br>❒ Natural frequency with different reference value	❒ yes<br>❒ no<br>❒ Indication on missing evidence	
**18**	Relative risk reduction/ relative risk excess / odds ratio / standardized mortality ratio	❒ yes<br>❒ no	❒yes<br>❒ no<br>❒ unclear	❒ Text<br>❒ Number<br>❒ Diagram<br>❒Table	❒ Percentage<br>❒ Absolute frequency without reference value<br>❒ Natural frequency with equal reference value<br>❒ Natural frequency with different reference value	❒ yes<br>❒ no<br>❒ Indication on missing evidence	
**19**	Number needed to screen / number needed to detect / number needed to treat	❒ yes<br>❒ no	❒yes<br>❒ no<br>❒ unclear	❒ Text<br>❒ Number<br>❒ Diagram<br>❒Table	❒ Percentage<br>❒ Absolute frequency without reference value<br>❒ Natural frequency with equal reference value<br>❒ Natural frequency with different reference value	❒ yes<br>❒ no<br>❒ Indication on missing evidence	
**Follow-up diagnostic**							
**20**	Follow-up diagnostic in case of a positive result	❒ yes<br>❒ no	❒yes<br>❒ no<br>❒ unclear			❒ yes<br>❒ no<br>❒ Indication on missing evidence	
**21**	Time of next PSA test in case of a negative result	❒ yes<br>❒ no	❒yes<br>❒ no<br>❒ unclear			❒ yes<br>❒ no<br>❒ Indication on missing evidence	
**Overdiagnosis**							
**22**	Possible overdiagnosis/ overtherapy	❒ yes<br>❒ no	❒yes<br>❒ no<br>❒ unclear			❒ yes<br>❒ no<br>❒ Indication on missing evidence	

	Criteria	Included?	Correct?	Kind of presentation?	Kind of numbers?	Strength of evidence indicated?	Quotes, Comments
**23**	Risk/number of screens of overdiagnosis/ overtherapy	❒ yes<br>❒ no	❒yes<br>❒ no<br>❒ unclear	❒ Text<br>❒ Number<br>❒ Diagram<br>❒Table	❒ Percentage<br>❒ Absolute frequency without reference value<br>❒ Natural frequency with equal reference value<br>❒ Natural frequency with different reference value	❒ yes<br>❒ no<br>❒ Indication on missing evidence	
**24**	Relative risk of prostate cancer incidence of screening compared to control group	❒ yes<br>❒ no	❒yes<br>❒ no<br>❒ unclear	❒ Text<br>❒ Number<br>❒ Diagram<br>❒Table	❒ Percentage<br>❒ Absolute frequency without reference value<br>❒ Natural frequency with equal reference value<br>❒ Natural frequency with different reference value	❒ yes<br>❒ no<br>❒ Indication on missing evidence	
**Risks of follow-up diagnostic (biopsy)**							
**25**	Risk of follow-up diagnostic	❒ yes<br>❒ no	❒yes<br>❒ no<br>❒ unclear	❒ Text<br>❒ Number<br>❒ Diagram<br>❒Table	❒ Percentage<br>❒ Absolute frequency without reference value<br>❒ Natural frequency with equal reference value<br>❒ Natural frequency with different reference value	❒ yes<br>❒ no<br>❒ Indication on missing evidence	
**26a**	Risk of bleeding	❒ yes<br>❒ no	❒yes<br>❒ no<br>❒ unclear	❒ Text<br>❒ Number<br>❒ Diagram<br>❒Table	❒ Percentage<br>❒ Absolute frequency without reference value<br>❒ Natural frequency with equal reference value<br>❒ Natural frequency with different reference value	❒ yes<br>❒ no<br>❒ Indication on missing evidence	
**26b**	Risk of pain	❒ yes<br>❒ no	❒yes<br>❒ no<br>❒ unclear	❒ Text<br>❒ Number<br>❒ Diagram<br>❒Table	❒ Percentage<br>❒ Absolute frequency without reference value<br>❒ Natural frequency with equal reference value<br>❒ Natural frequency with different reference value	❒ yes<br>❒ no<br>❒ Indication on missing evidence	
**26c**	Risk of infection	❒ yes<br>❒ no	❒yes<br>❒ no<br>❒ unclear	❒ Text<br>❒ Number<br>❒ Diagram<br>❒Table	❒ Percentage<br>❒ Absolute frequency without reference value<br>❒ Natural frequency with equal reference value<br>❒ Natural frequency with different reference value	❒ yes<br>❒ no<br>❒ Indication on missing evidence	
**26d**	Risk of impotence	❒ yes<br>❒ no	❒yes<br>❒ no<br>❒ unclear	❒ Text<br>❒ Number<br>❒ Diagram<br>❒Table	❒ Percentage<br>❒ Absolute frequency without reference value<br>❒ Natural frequency with equal reference value<br>❒ Natural frequency with different reference value	❒ yes<br>❒ no<br>❒ Indication on missing evidence	
**26e**	Risk of incontinence	❒ yes<br>❒ no	❒yes<br>❒ no<br>❒ unclear	❒ Text<br>❒ Number<br>❒ Diagram<br>❒Table	❒ Percentage<br>❒ Absolute frequency without reference value<br>❒ Natural frequency with equal reference value<br>❒ Natural frequency with different reference value	❒ yes<br>❒ no<br>❒ Indication on missing evidence	
**26f**	Risk of psychological distress	❒ yes<br>❒ no	❒yes<br>❒ no<br>❒ unclear	❒ Text<br>❒ Number<br>❒ Diagram<br>❒Table	❒ Percentage<br>❒ Absolute frequency without reference value<br>❒ Natural frequency with equal reference value<br>❒ Natural frequency with different reference value	❒ yes<br>❒ no<br>❒ Indication on missing evidence	
**26g**	Risk of mortality	❒ yes<br>❒ no	❒yes<br>❒ no<br>❒ unclear	❒ Text<br>❒ Number<br>❒ Diagram<br>❒Table	❒ Percentage<br>❒ Absolute frequency without reference value<br>❒ Natural frequency with equal reference value<br>❒ Natural frequency with different reference value	❒ yes<br>❒ no<br>❒ Indication on missing evidence	
**Risk of prostate cancer treatment**							
**27**	Risks of PCa treatment (hormone therapy, surgery, radiotherapy, chemotherapy) (risks that cannot be assigned to the other points)	❒ yes<br>❒ no	❒yes<br>❒ no<br>❒ unclear	❒ Text<br>❒ Number<br>❒ Diagram<br>❒Table	❒ Percentage<br>❒ Absolute frequency without reference value<br>❒ Natural frequency with equal reference value<br>❒ Natural frequency with different reference value	❒ yes<br>❒ no<br>❒ Indication on missing evidence	
**28a**	Risk of urinary problems / incontinence	❒ yes<br>❒ no	❒yes<br>❒ no<br>❒ unclear	❒ Text<br>❒ Number<br>❒ Diagram	❒ Percentage<br>❒ Absolute frequency without reference value<br>❒ Natural frequency with equal reference value	❒ yes<br>❒ no<br>❒ Indication on missing evidence	

	Criteria	Included?	Correct?	Kind of presentation?	Kind of numbers?	Strength of evidence indicated?	Quotes, Comments
				❒Table	❒ Natural frequency with different reference value		
**28b**	Risk of impotence	❒ yes<br>❒ no	❒yes<br>❒ no<br>❒ unclear	❒ Text<br>❒ Number<br>❒ Diagram<br>❒Table	❒ Percentage<br>❒ Absolute frequency without reference value<br>❒ Natural frequency with equal reference value<br>❒ Natural frequency with different reference value	❒ yes<br>❒ no<br>❒ Indication on missing evidence	
**28c**	Risk of mortality	❒ yes<br>❒ no	❒yes<br>❒ no<br>❒ unclear	❒ Text<br>❒ Number<br>❒ Diagram<br>❒Table	❒ Percentage<br>❒ Absolute frequency without reference value<br>❒ Natural frequency with equal reference value<br>❒ Natural frequency with different reference value	❒ yes<br>❒ no<br>❒ Indication on missing evidence	
**Psychological distress caused by PCa diagnosis**							
**29**	Incidence of suicide; Standardized mortality ratio after PCa diagnosis	❒ yes<br>❒ no	❒yes<br>❒ no<br>❒ unclear	❒ Text<br>❒ Number<br>❒ Diagram<br>❒Table	❒ Percentage<br>❒ Absolute frequency without reference value<br>❒ Natural frequency with equal reference value<br>❒ Natural frequency with different reference value	❒ yes<br>❒ no<br>❒ Indication on missing evidence	
**Psychological distress caused by false-positive results**							
**30**	Risk of psychological distress caused by false-positive results	❒ yes<br>❒ no	❒yes<br>❒ no<br>❒ unclear	❒ Text<br>❒ Number<br>❒ Diagram<br>❒Table		❒ yes<br>❒ no<br>❒ Indication on missing evidence	
**31a**	Risk of anxiety and worry	❒ yes<br>❒ no	❒yes<br>❒ no<br>❒ unclear	❒ Text<br>❒ Number<br>❒ Diagram<br>❒Table		❒ yes<br>❒ no<br>❒ Indication on missing evidence	
**31b**	Risk of sexual dysfunction	❒ yes<br>❒ no	❒yes<br>❒ no<br>❒ unclear	❒ Text<br>❒ Number<br>❒ Diagram<br>❒Table		❒ yes<br>❒ no<br>❒ Indication on missing evidence	
**31c**	Risk of impaired quality of life	❒ yes<br>❒ no	❒yes<br>❒ no<br>❒ unclear	❒ Text<br>❒ Number<br>❒ Diagram<br>❒Table		❒ yes<br>❒ no<br>❒ Indication on missing evidence	
**Others**							
**32**	Years of Life Lost	❒ yes<br>❒ no	❒yes<br>❒ no<br>❒ unclear	❒ Text<br>❒ Number<br>❒ Diagram<br>❒Table	❒ Percentage<br>❒ Absolute frequency without reference value<br>❒ Natural frequency with equal reference value<br>❒ Natural frequency with different reference value	❒ yes<br>❒ no<br>❒ Indication on missing evidence	
**33**	Years of Life Gained	❒ yes<br>❒ no	❒yes<br>❒ no<br>❒ unclear	❒ Text<br>❒ Number<br>❒ Diagram<br>❒Table	❒ Percentage<br>❒ Absolute frequency without reference value<br>❒ Natural frequency with equal reference value<br>❒ Natural frequency with different reference value	❒ yes<br>❒ no<br>❒ Indication on missing evidence	
